# Dynamic transcriptome changes during osteogenic differentiation of bone marrow-derived mesenchymal stem cells isolated from chicken

**DOI:** 10.3389/fcell.2022.940248

**Published:** 2022-09-02

**Authors:** Huijiao Lv, Tao Wang, Shangkun Zhai, Zhuocheng Hou, Sirui Chen

**Affiliations:** National Engineering Laboratory for Animal Breeding and MARA Key Laboratory of Animal Genetics, Breeding and Reproduction, College of Animal Science and Technology, China Agricultural University, Beijing, China

**Keywords:** bone marrow mesenchymal stem cells, osteoblastic differentiation, RNA-seq transcriptome analysis, differentially expressed gene, chicken

## Abstract

Osteoblasts are indispensable for skeletal growth and maintenance. Bone marrow-derived mesenchymal stem cells (BMSCs) are useful in studying osteogenesis. In this study, BMSCs isolated from White Leghorns were differentiated into osteoblasts *in vitro*. Cells induced for -1, 0, 1, 11, and 22 d were used for transcriptomic analyses using the HISAT2-Stringtie-DESeq2 pipeline. Weighted correlation network analysis was processed to investigate significant modules, including differentially expressed genes (DEGs), correlated with osteogenic differentiation. Gene ontology and pathway enrichment analyses of DEGs were performed to elucidate the mechanisms of osteoblast differentiation. A total of 534, 1,144, 1,077, and 337 DEGs were identified between cells induced for -1 and 0, 0 and 1, 1 and 11, and 11 and 22 d, respectively (|log2FC| > 1.0, FDR <0.05). DEGs were mainly enriched in pathways related to cell proliferation in the early stage of osteogenic differentiation and pathways, such as the TGF-β signaling pathway, in the middle and late stages of osteogenic differentiation. A protein–protein interaction network of the 87 DEGs in the MEturquoise module within top 5-%-degree value was built utilizing the STRING database. This study is the first to elucidate the transcriptomic changes in the osteogenic differentiation of BMSCs isolated from White Leghorns at different times. Our results provide insight into the dynamic transcriptome changes during BMSC differentiation into osteoblasts in chicken.

## Introduction

Commercially bred chickens are reared in high-density feeding environments to meet the demand for meat and egg production (Stratmann et al., 2015). Rapid weight gain (Bradshaw et al., 2002) and prolonged lack of exercise (Webster, 2004; Lay et al., 2011) increase the burden on the chicken skeletal system, leading to abnormal bone development, fractures, and even death. Skeletal problems in poultry are particularly important economic and animal welfare concerns. Therefore, bone properties are important indicators used for evaluating poultry quality and cultivating new poultry varieties in many countries (Rubin et al., 2007; Gonzalez-Ceron et al., 2015).

Bone is composed of a mineral matrix, mainly hydroxyapatite, and an organic matrix containing collagen, lipids, proteoglycans, and other bone structural proteins (Johnsson et al., 2015). A reduction of the mineralized bone mass increases the incidence of bone fragility and fractures in chickens, similar to humans and mammals (Rubin et al., 2007). Previous studies have noted that the skeletal system of chickens has a complex phenotype influenced by genetics, nutrition, hormones, and housing type (Reid, 1998; Rath et al., 2000; Whitehead & Fleming, 2000). Among these factors, genetic factors are the most fundamental (Rubin et al., 2007; Gonzalez-Ceron et al., 2015).

Osteoblasts account for 4–6% of the total residential bone cells and maintain bone mass by interacting with osteoclasts and osteocytes (Capulli et al., 2014). Osteoblasts derived from pluripotent mesenchymal stem cells (MSCs) have self-renewal capacity (Shanti et al., 2007) and can be obtained from different origins, such as bone marrow and adipose tissue (Beane & Darling, 2012). Current studies on bone marrow-derived MSCs (BMSCs) mostly use cells isolated from mammals, such as humans (Baghaei et al., 2017), rats (Li et al., 2013), mice (Huang et al., 2015), and pigs (Feyen et al., 2016). Chicken BMSCs have been successfully isolated from chicken embryos (Young et al., 1993) and postnatal chicken bone marrow (Bai et al., 2013). Alkaline phosphatase (ALP) staining was positive and alizarin red staining demonstrated the formation of mineralized calcium nodules when the osteogenic differentiation of BMSCs into osteoblasts was induced *in vitro*.

To date, studies on chicken skeletal system development and gene regulation mechanisms have been incomplete. In this study, dynamic changes in the transcriptome during osteogenic differentiation of BMSCs isolated from White Leghorn chicken embryos incubated for 15 d were analyzed. To our knowledge, this is the first study to analyze osteogenic differentiation of chicken BMSCs using transcriptome sequencing. Therefore, our study contributes a novel approach for further research on bone development in chicken by improving the gene regulatory network.

## Materials and methods

Unless otherwise stated, all chemicals used in the experiments were purchased from Sigma-Aldrich (St. Louis, MO, USA). All animal experiments followed the principles formulated by the Ministry of Agriculture of China and were performed in accordance with the guidelines of the Animal Care Committee of China Agricultural University (permit number: AW01111202-1-2).

### Isolation and culture of BMSCs

White Leghorn eggs, hatched after incubation in an environment with a temperature of 37°C, 5% CO2, and 60% humidity for 15 d, were provided by Poultry Genetic Resources and Breeding Experimental Base of China Agricultural University. After euthanasia, the thighs were separated from the chicken embryos and soaked in 75% alcohol for 5 min under sterile conditions. The tibia was obtained after removing the attached muscle tissue and rinsing with phosphate-buffered saline (PBS) containing 5% penicillin-streptomycin (Gibco) three times. The metaphyseal was resected, and bone marrow was collected from the tibia by flushing with complete medium containing DMEM/F-12 (Gibco), 10% fetal bovine serum (FBS) (Gibco), and 1% penicillin-streptomycin (Gibco). The cell suspension was then centrifuged and cells were washed three times at 100 *g* for 10 min in complete medium. Then, the cells were resuspended in complete medium and plated into a 25 cm^2^ culture flask (Corning, Glendale, AZ, USA). The medium was refreshed for the first time after 14 h and every 2 days thereafter.

### Purification and analyses of cell surface markers

Cells were subcultured at a ratio of 1:2 when the cell confluence reached approximately 80% and purified using the differential adhesion method. After dissociation with 0.25% trypsin-EDTA (Gibco) and centrifugation at 100 *g* for 5 min, cells were suspended in complete medium and plated into culture flasks. Nonadherent cells were removed after 30 min and replaced with fresh medium. Adherent cells were labeled as the P1 generation. The procedure was repeated and P2, P3,..., Pn generations were obtained for further experiments.

Generation P3 was used for immunofluorescence staining and RT-PCR analysis of cell surface markers. Immunofluorescence assays was adopted to analyzed the expressions of CD29, CD44 and CD45. Cells were plated and fixed with 4% paraformaldehyde (Biosharp, Hefei, Anhui, China) and penetrated with 0.5% Triton X-100 (Solarbio, Beijing, China). The detections of P3 BMSCs CD29 (Proteintech, Wuhan, Hubei, China), CD44 (Bioss, Beijing, China), and CD45 (Proteintech, Wuhan, Hubei, China) were performed according to the instructions of antibodies. The nucleus was stained with DAPI (Solarbio, Beijing, China). Total RNA was extracted from P3 BMSCs using the Total RNA Extraction Kit (Omega BioTek, Norcross, GA, USA), and cDNA was obtained using the PrimeScript RT reagent Kit (TaKaRa Bio, Shiga, Japan) following the manufacturer’s manual. Primers for CD29, CD44, CD45, CD71, and GAPDH were specifically designed for PCR amplification (Table 1).

**TABLE 1:** The primers for BMSCs identification.

Genes	Primer sequences (5′–3′)	Product length (bp)	Tm (°C)
CD29	F: GAA​CGG​ACA​GAT​ATG​CAA​CGG	300	62
	R: TAG​AAC​CAG​CAG​TCA​CCA​ACG		
CD44	F: GGT​TTT​ATA​GTG​GGG​CAT​ATT​GTT​ATC​CC	700	62
	R: TTA​ACC​GCG​ATG​CAC​ACG​GC		
CD71	F: CCCAGGCTTCCCTTCGT	310	55
	R: GGG​CTC​CAA​TCA​CAA​CAT​AC		
CD45	F: CAC​TGG​GAA​TCG​AGA​GGA​AA	574	55
	R: CTG​GTC​TGG​ATG​GCA​CTT​TT		
GAPDH	F: TAA​AGG​CGA​GAT​GGT​GAA​AG	244	58
	R: ACG​CTC​CTG​GAA​GAT​AGT​GAT		

F, forward; R, reverse.

### Induction and detection of osteogenic differentiation *in vitro*

Generation P3 BMSCs were plated in 12-well plates and 75 cm^2^ culture flasks for osteogenic differentiation. Osteogenic induction medium, containing DMEM/F-12, 10% FBS, 1% penicillin-streptomycin, 10 nmol/L dexamethasone, 10 mmol/L β-glycerophosphate, and 50 μg/mL L-ascorbic acid, was added to the osteogenic induction groups to induce osteogenic differentiation of BMSCs. BMSCs at a cell confluence of 80% were labeled as the -1 day group, for studying changes in gene expression profile between varying stages of proliferation and levels of contact inhibition. After 24 h, BMSCs at a cell confluence of approximately 90% were labeled as the day zero group. A total of five osteogenic induction groups were set in this experiment depending on the number of days of osteogenic induction (-1, 0, 1, 11, and 22 d). The induction experiments were replicated three times for each group. During osteogenic differentiation, the induction medium was replaced every 3 days. Cells in culture flasks were collected for mRNA-seq on the corresponding days of induction. Concurrently, cells from these osteogenic induction groups in the 12-well plates were used for ALP staining (Beyotime Biotechnology, Shanghai, China) and Alizarin Red staining (Solarbio, Beijing, China), according to the manufacturer’s instructions.

### RNA extraction and mRNA sequencing

Cells from the different treatment groups were collected in 2-ml tubes. The total RNA of each sample was extracted using a Total RNA Kit (Omega) according to the manufacturer’s instructions. The quality and concentration of the RNA samples were confirmed using NanoDrop-2000 (Thermo Fisher Scientific, Waltham, MA, USA). All RNA samples with ultraviolet absorbance ratios (A_260_/A_280_) between 1.8 and 2.0 were selected for RNA sequencing library preparation. cDNA library construction was performed using the Illumina NovaSeq PE150 platform (Beijing Youji Technology, Beijing, China). Clean reads used for further analysis were filtered and generated according to the following criteria: 1) reads with adapter pollution or N ratio of >5%; 2) reads with low quality of Q-value <20. The RNA-seq raw data were deposited in the NCBI SRA database under the accession number PRJNA824833.

### Bioinformatics analysis

Read mapping and transcript expression level quantification were performed using the HISAT2, StringTie, and DESeq2 workflows (Pertea et al., 2016). The high-quality reads obtained were aligned to the chicken reference genome (GRC6a) using HISAT2 (v2.1.0) (Kim et al., 2015). Transcript assembly, GTF document merging, and transcript quantification were performed using StringTie (v1.3.5) (Pertea et al., 2015). Statistical analysis of DEGs was performed using DESeq2 (v) with the criteria of |log2FC| >1.0 and FDR <0.05. Gene ontology (GO) and Kyoto Encyclopedia of Genes and Genomes (KEGG) pathway analyses of DEGs were performed using DAVID (https://david.ncifcrf.gov/home.jsp) with default parameters. Weighted correlation network analysis (WGCNA) (Langfelder & Horvath, 2008) was performed to identify the modules of highly correlated genes during osteogenic differentiation. STRING (https://string-db.org/) and CytoNCA (Tang et al., 2015) were used to analyze and visualize the protein–protein interaction network of genes in the identified modules with high correlation.

## Results

### Purification and identification of BMSCs

**FIGURE 1:**
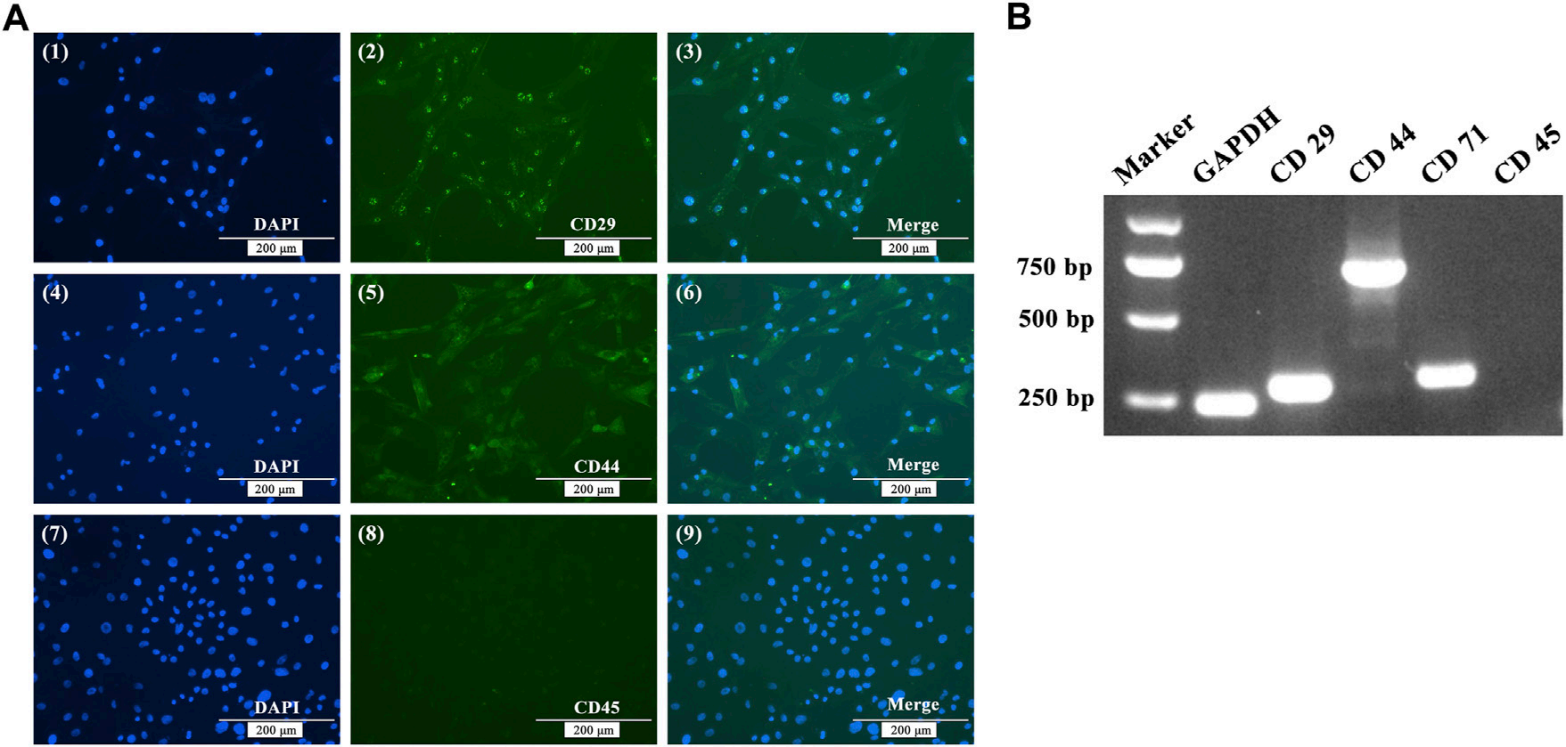
Immunofluorescence staining **(A)** and RT-PCR **(B)** analysis of BMSC cell surface markers. **(A)** Immunofluorescence showing positive staining for CD29 and CD44, but negative for CD45. **(B)** RT-PCR assays showed that the expression of GADPH, CD29, CD44 and CD71 were positive, while the expression of CD45 was negative.

After using the differential adhesion method for BMSC purification three times, immunofluorescence staining was applied to detected cell surface markers. Immunofluorescence showed that BMSCs were positive staining for CD29 and CD44, but negative for CD45 (Figure 1A). RNA was extracted from P3 BMSCs, reverse transcribed to cDNA, and RT-PCR was used to identify BMSCs. The results showed that P3 BMSCs isolated from White Leghorns were positive for CD29, CD44, and CD71, but negative for CD45 (Figure 1B).

### Osteogenic induction of BMSCs *in vitro*

**FIGURE 2:**
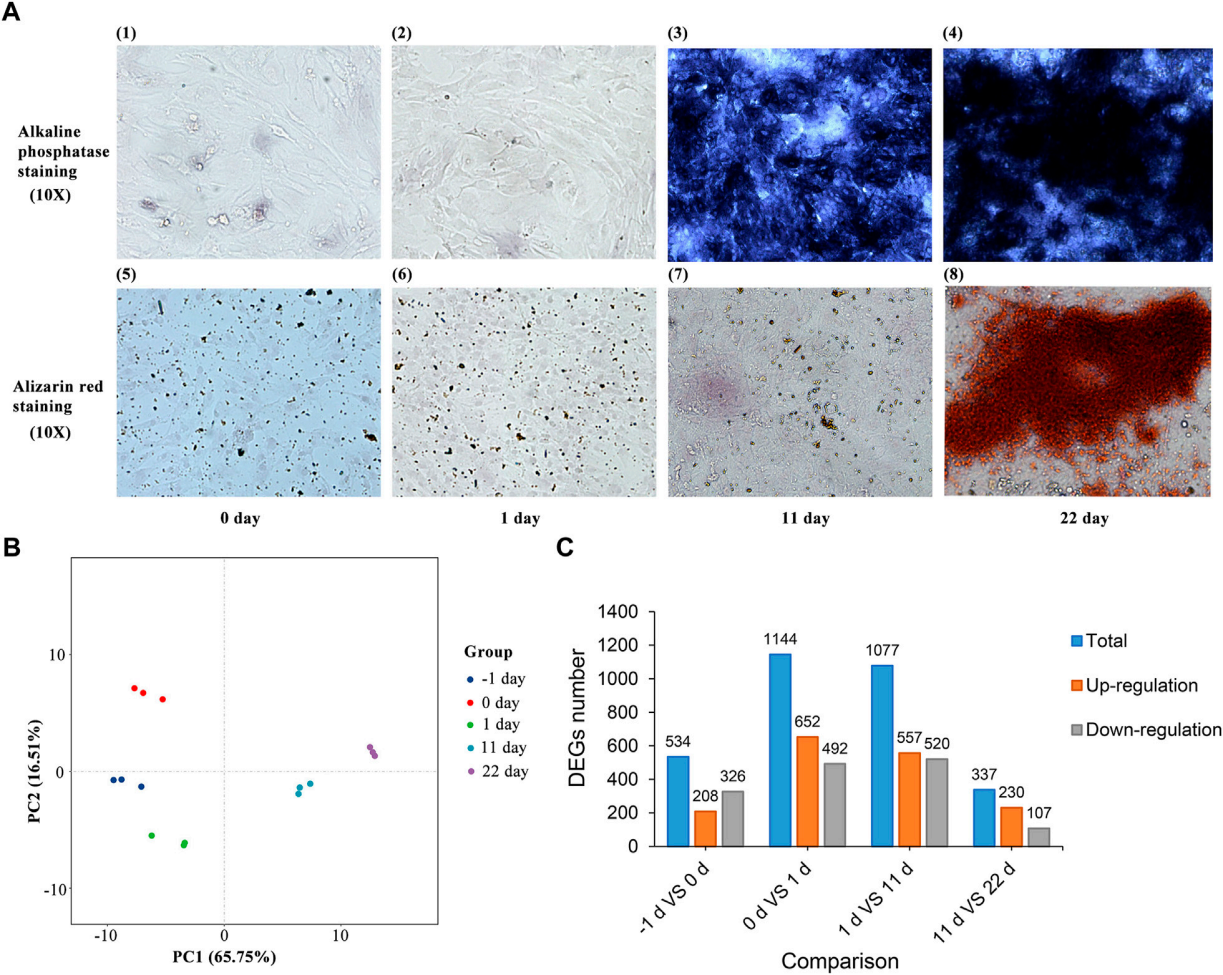
**(A)** ALP staining and Alizarin Red staining of BMSC osteogenic induction. **(B)** Principal component analysis of BMSCs induced for -1, 0, 1, 11 and 22 d. **(C)** Statistics of the differentially expressed genes between adjacent induction groups.

Under the same experimental conditions, Alizarin Red staining and ALP staining were performed on BMSCs after osteogenic differentiation in a 12-well plate at different time points. As shown in Figure 2A, ALP staining and Alizarin Red staining of BMSCs were detected 22 d after induction. These results suggest that chicken BMSCs successfully differentiated into osteoblasts.

### Transcriptome dynamics during osteogenic differentiation of BMSCs and functional annotation of DEGs

After processing with StringTie, principal component analysis (PCA) of all the samples was performed (Figure 2B). The results showed that differences between and within groups were appropriate for subsequent analysis. DEGs between groups with different induction times were identified after analysis using DESeq2. A total of 534, 1,144, 1,077, and 337 DEGs (Figure 2C and Supplementary Table S1) were identified between the groups induced for -1 and 0, 0 and 1, 1 and 11, and 11 and 22 d, respectively (|log2FC| > 1.0, FDR <0.05).

**FIGURE 3:**
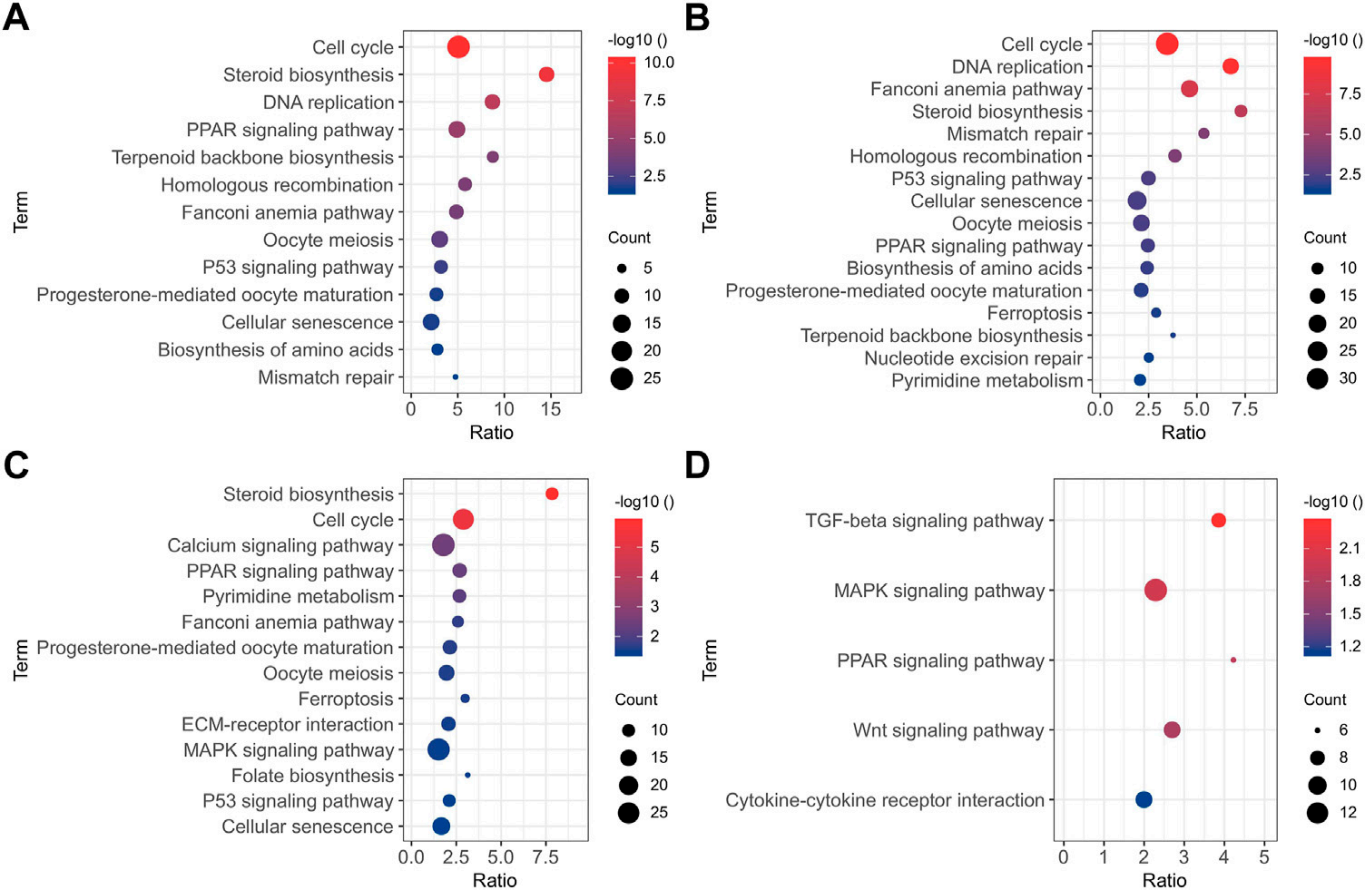
KEGG pathway analysis (*p* < 0.05) of DEGs between 0 and -1 **(A)**, 1 and 0 **(B)**, 11 and 1 **(C)**, and 22 and 11 **(D)** d of osteogenic induction.

All the DEGs identified in the present study may influence the osteogenic differentiation of BMSCs. Functional annotation was performed on all DEGs to ascertain the functions of these genes. The results of the GO enrichment analysis and KEGG pathway analysis of up-regulated and down-regulated DEGs between two adjacent groups are displayed in Supplementary Table S2 and Supplementary Table S3, respectively. To explicitly show all the DEGs at the different times of the osteogenesis induction, the results of the KEGG pathway (*p* < 0.05) are presented in Figure 3. In the early stage of osteogenic differentiation (Figures 3A,B), the DEGs were enriched in pathways including the cell cycle, DNA replication, homologous recombination, and mismatch repair. The DEGs between 11 and 1 d of osteogenic differentiation (Figure 3C) were enriched in steroid biosynthesis, cell cycle, calcium signaling pathway, ECM–receptor interaction, and the MAPK signaling pathway. The DEGs between 22 and 11 d of osteogenic differentiation (Figure 3D) were mostly enriched in the TGF-β signaling pathway, MAPK signaling pathway, PPAR signaling pathway, Wnt signaling pathway, and cytokine–cytokine receptor interaction.

### WGCNA of osteogenic differentiation

**FIGURE 4:**
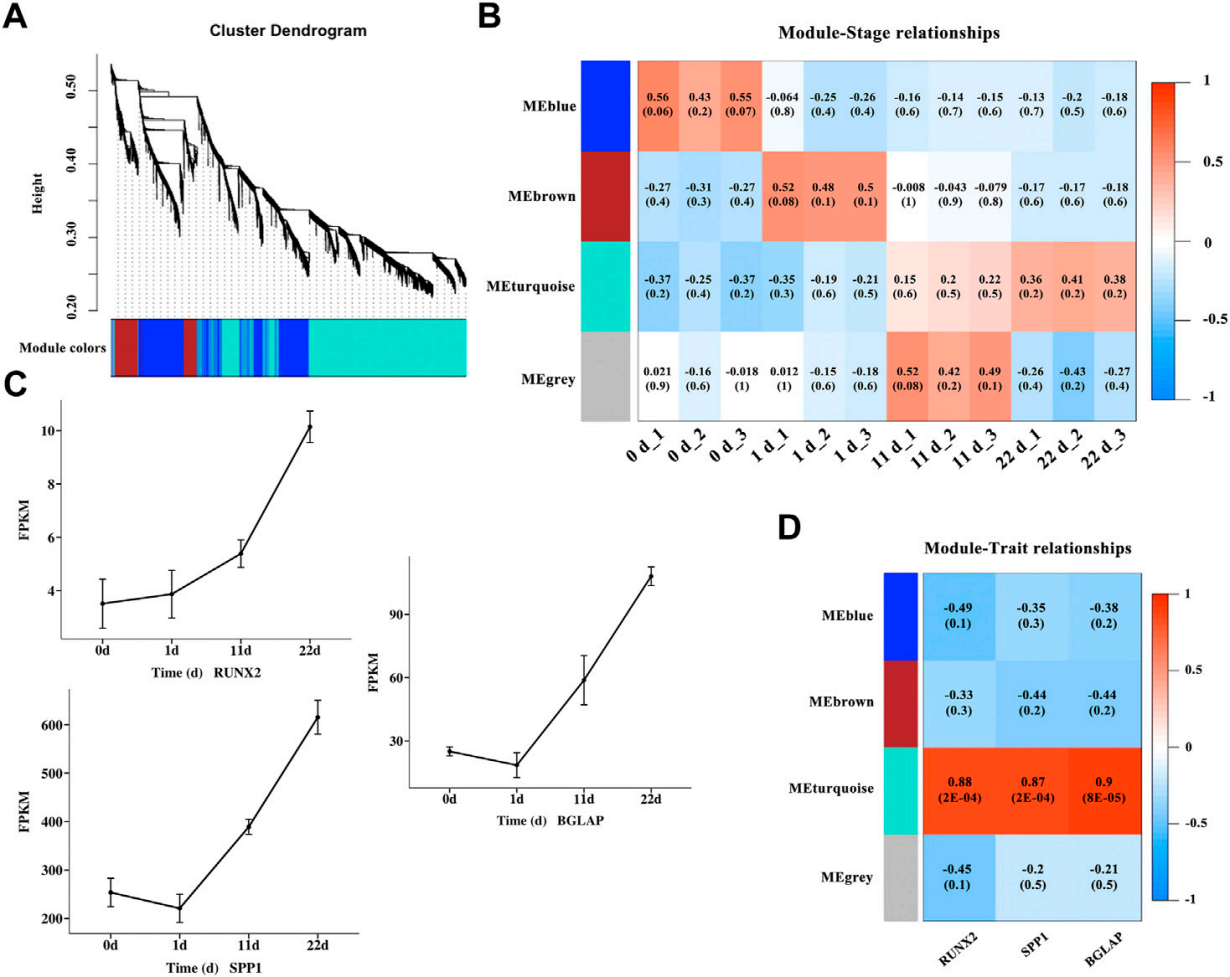
Identification of modules associated with osteogenic differentiation in different induction stages. **(A)** Clustering dendrogram of DEGs in different osteogenic induction groups. As a result, 4 modules with different colors were constructed. **(B)** Module-trait association and the corresponding *p*-value with all the DEGs (listed in Supplementary Table S4) at different osteogenic induction time. **(C)** The mRNA variation tendency of genes RUNX2, SPP1 and BGLAP. Horizontal coordinate represents the induction time. The ordinate represents the changes of fragments per kilobase per million (FPKM). **(D)** Module-gene correlations and the corresponding *p*-value of genes *RUNX2*, *SPP1* and *BGLAP*.

To gain a deeper understanding of the gene interactions occurring during osteogenic differentiation of BMSCs, WGCNA was performed to identify significant modules including DEGs that are highly correlated to osteogenic differentiation. A total of 4,577 nonredundant DEGs (Supplementary Table S4) was used to evaluate availability and construct a gene co-expression network (Figure 4A). The results of module-trait relationships (Figure 4B) showed that the MEblue module on day zero of induction, genes in the MEbrown module on day one of induction, and genes in the MEturquoise module at 11 and 22 days of induction were correlated with osteogenic differentiation, indicating that the MEblue and MEbrown modules are related with the early stage of osteogenic differentiation and that the MEturquoise module is related with the middle and late stages of osteogenic differentiation. The gene sets in the above induction times and corresponding modules were extracted for GO enrichment (Supplementary Table S5) and KEGG pathway (Table 2) analyses. The results of KEGG pathway analysis showed that the genes in the early stage of osteogenic differentiation were mainly enriched in pathways related to cell proliferation, such as DNA replication (gga03030), homologous recombination (gga03440), and cell cycle (gga04110), and the corresponding genes in the middle and late stages of osteogenic differentiation were mainly enriched in the PPAR signaling pathway (gga03320), MAPK signaling pathway (gga04010), TGF-β signaling pathway (gga04350), Wnt signaling pathway (Wnt signaling pathway), and others. The expression of the genes *RUNX2*, *SPP1*, and *BGLAP* (Figure 4C) and the correlation between genes and modules (Figure 4D) were analyzed, and the results showed that the turquoise module was significantly correlated (0.87–0.9, *p*-value < 0.01) with these three osteogenic marker genes.

**TABLE 2:** KEGG pathway analysis of genes at the different induction times and corresponding modules.

Stage	KEGG pathway	*p*-value	Genes
DEGs at 0 d	DNA replication (gga03030)	2.42E-08	RFC3, RFC2, MCM3, MCM4, MCM5, MCM6, DNA2, POLE, MCM2
	Cell cycle (gga04110)	1.15E-02	RBL1, MCM3, MCM4, MCM5, TTK, MCM6, MCM2
	Mismatch repair (gga03430)	4.61E-02	RFC3, EXO1, RFC2
	Homologous recombination (gga03440)	6.89E-02	RAD51B, RAD52, RAD54L
	Retinol metabolism (gga00830)	9.46E-02	ALDH1A2, AOX2, RDH5
DEGs at 1 d	Cell cycle (gga04110)	1.44E-12	PCNA, PLK1, BUB1B, CDC7, CDC6, CCNB3, CCNA2, ORC5, CDC20, WEE1, CCNE2, ESPL1, ORC1, CHEK1, CDK2, E2F1, CDK1, BUB1, MAD2L1
	Steroid biosynthesis (gga00100)	1.47E-08	SQLE, NSDHL, CYP51A1, SC5D, MSMO1, DHCR24, LSS, FDFT1
	Fanconi anemia pathway (gga03460)	1.79E-08	BLM, RAD51, EME1, RMI1, UBE2T, RPA2, FAAP24, FANCC, FANCB, BRCA2, FANCG
	Biosynthesis of antibiotics (gga01130)	9.14E-06	FDPS, MVK, ACAA2, CYP51A1, MSMO1, HMGCR, ENO1, LSS, ACAT2, SQLE, ACLY, NSDHL, SC5D, MVD, FDFT1
	Oocyte meiosis (gga04114)	5.04E-05	CDC20, CCNE2, ESPL1, PLK1, CDK2, CDK1, FBXO5, BUB1, AURKA, MAD2L1
	Homologous recombination (gga03440)	1.02E-04	BLM, RAD51, EME1, RPA2, BRCA2, RAD54B
	Terpenoid backbone biosynthesis (gga00900)	6.45E-04	FDPS, MVK, MVD, HMGCR, ACAT2
	p53 signaling pathway (gga04115)	1.13E-03	CCNB3, RRM2, CCNE2, CHEK1, CDK2, CDK1, GTSE1
	DNA replication (gga03030)	2.58E-03	PRIM2, FEN1, PCNA, LIG1, RPA2
	Progesterone-mediated oocyte maturation (gga04914)	3.29E-03	CCNB3, CCNA2, PLK1, CDK2, CDK1, BUB1, MAD2L1
	Metabolic pathways (gga01100)	1.59E-02	PRIM2, ACSS3, ACAA2, MVK, DGKB, MSMO1, HMGCR, ENO1, ACAT2, DGUOK, NSDHL, B3GALT2, LIPG, SC5D, TK1, FDFT1, FDPS, DUT, RRM2, MMAB, CYP51A1, ACSL5, DHCR24, LSS, DCK, DHFR, SQLE, ACLY, AMACR, P4HA2, MVD
	Pyrimidine metabolism (gga00240)	2.62E-02	PRIM2, DGUOK, DUT, RRM2, TK1, DCK
	Fatty acid metabolism (gga01212)	5.45E-02	FADS2, ACAA2, ACSL5, ACAT2
	Mismatch repair (gga03430)	5.77E-02	PCNA, LIG1, RPA2
DEGs at 11 d	PPAR signaling pathway (gga03320)	1.61E-03	FABP3, SLC27A1, FABP5, FABP7, ADIPOQ, LPL, CD36, PLIN1, PLTP, ACSBG2
	MAPK signaling pathway (gga04010)	2.11E-02	DUSP4, DUSP5, NTRK2, CACNA2D2, PDGFA, FOS, NGF, FGF1, RASGRP1, TGFBR2, RASGRP3, FGF7, PAK1, CACNB4, FGF9, FGFR3, CACNG3, FGF10
	ECM-receptor interaction (gga04512)	2.11E-02	LAMA5, VTN, VWF, COL4A2, COL4A1, COL6A2, COL6A1, ITGA8, CD36
	Neuroactive ligand-receptor interaction (gga04080)	2.20E-02	GRIA1, CHRNB2, GABRB2, UTS2R, GCGR, CHRNA7, PTGER3, HTR1A, HTR1B, ADRB2, LPAR4, OPRM1, SSTR2, ADRA2C, GRIN3B, GRIN3A, HTR7, BDKRB2, F2RL1, BDKRB1, S1PR3
	Steroid biosynthesis (gga00100)	4.37E-02	SOAT1, DHCR7, HSD17B7, LIPA
	TGF-β signaling pathway (gga04350)	5.96E-02	BMP4, CDKN2B, BMP2, ID1, ID4, NOG2, ACVR2B, TGFBR2
	Cell adhesion molecules (CAMs) (gga04514)	7.21E-02	NLGN3, CNTNAP1, ALCAM, NRXN1, SDC3, ITGA8, NRXN3, PTPRM, NCAM1, NRCAM
	Calcium signaling pathway (gga04020)	7.83E-02	RYR2, CHRNA7, PTGER3, ADRB2, RYR3, HTR7, ERBB4, CAMK4, BDKRB2, PPIF, PLCG2, BDKRB1, SLC25A4
DEGs at 22 d	TGF-β signaling pathway (gga04350)	8.25E-04	TGIF1, BMP2, BMPR2, ID1, ID3, ACVR2B, TGFBR2
	MAPK signaling pathway (gga04010)	4.33E-03	PDGFRB, PDGFRA, DUSP5, JUN, PAK1, DUSP1, IL1R1, FOS, DUSP8, TGFBR2
	PPAR signaling pathway (gga03320)	1.20E-02	FABP5, APOA1, ANGPTL4, CD36, PLIN1
	Cytokine-cytokine receptor interaction (gga04060)	2.30E-02	IL6, BMPR2, CXCL12, IL1R1, ACVR2B, CX3CL1, TGFBR2
	Wnt signaling pathway (gga04310)	3.42E-02	WNT6, JUN, MMP7, WNT5B, WNT9A, LRP6
	Focal adhesion (gga04510)	6.02E-02	PDGFRB, PDGFRA, JUN, PAK1, COL4A2, BCL2, ITGA9
	ECM-receptor interaction (gga04512)	9.95E-02	COL4A2, CD36, GP5, ITGA9

### Protein–protein interaction network

**FIGURE 5:**
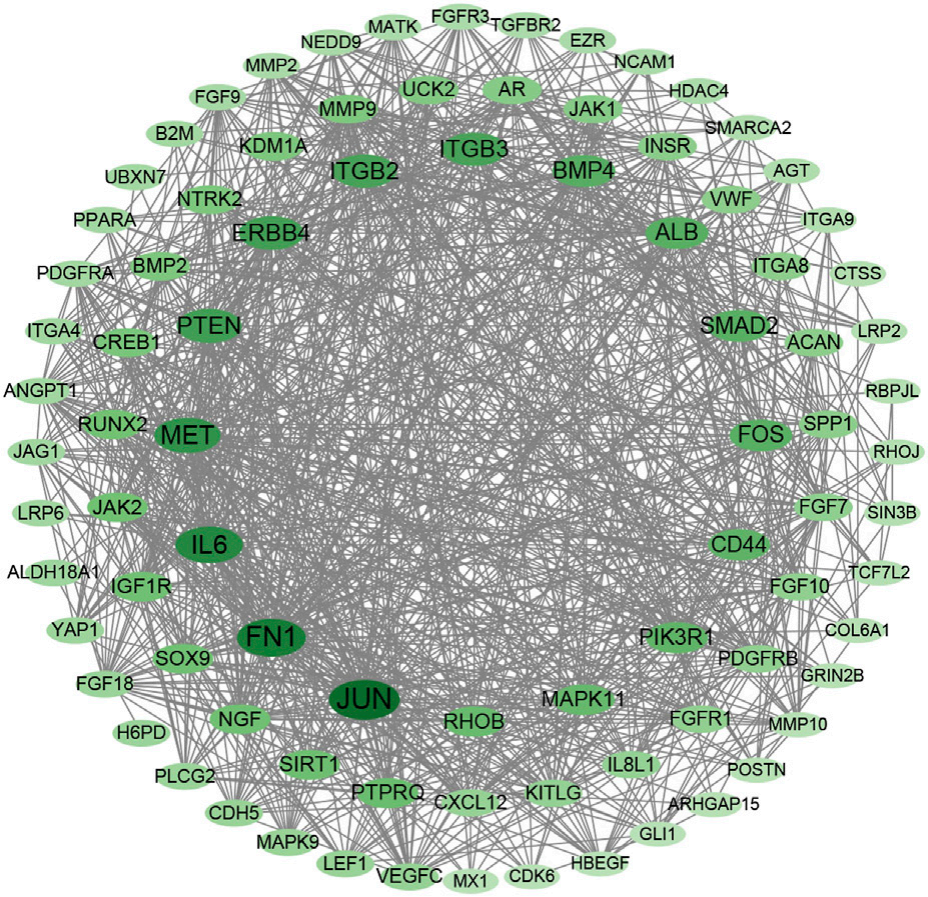
Protein–protein interaction network with DEGs in MEturquoise module within the top 5-%-degree value (Supplementary Table S6).

A protein–protein interaction (PPI) network was constructed for DEGs in the MEturquoise module with STRING database and Cytoscape. A total of 87 nodes with the top 5%-degree value calculated by CytoNCA (Supplementary Table S6) were mapped to the PPI network (Figure 5), including JUN, FN1, IL6, MET, PTEN, ERBB4, ITGB2, ITGB3, BMP4, RUNX2, and SPP1.

## Discussion

Over the long process of domestication, artificial selection has led to phenotypic differences between domestic chickens and *Gallus* (Talaty et al., 2010). The bone health of commercial breeds of laying hens (Hocking et al., 2004) and broilers (Silversides et al., 2006) varies considerably and the bone of these breeds is generally weaker than that of traditional breeds. Osteoporosis and fractures worsen the welfare and economic concerns in the chicken industry. Bone maturation involves various complicated molecular and biochemical changes in osteoblasts and osteoclasts. After basic structural development and mineralization are completed, the mature bone has physical and biological mechanical properties (Rath et al., 2000). Factors including bone mineral density, bone microarchitecture, bone geometry, and bone material properties are relevant to bone strength (Friedman, 2006). Studies have shown that the development of the skeletal system, like that of other systems and traits, is controlled by complex genetic networks (Bishop et al., 2000; Schreiweis et al., 2005; Raymond et al., 2018). Genetic selection has significant potential for improving bone strength and reducing osteoporosis (Mandour et al., 1989). Understanding the mechanisms of bone metabolism, such as mineralization during bone development, will provide novel insights into improving poultry bone performance and industrial efficiency in the future.

BMSCs have the potential for multi-lineage differentiation, such as osteogenic and adipogenic differentiation, making them useful tools for studying mineralization during osteogenesis. Many studies have demonstrated the potential of BMSCs for treating osteoporosis and osteoarthritis in humans (Kiernan et al., 2016; Liu et al., 2021). However, collection of human BMSCs requires general anesthesia, limiting its research and clinical applications (Mushahary et al., 2018). Based on previous research, MSCs from other tissues, such as adipose tissues and dental tissues, have become viable substitutes. At present, research on BMSCs in humans and mice mostly focus on the function of one or several genes (Frank et al., 2002; Wang et al., 2021) or microRNA (Lin et al., 2019) in the process of osteogenic differentiation. Zhang et al. analyzed the functional network of differential long noncoding RNA/mRNA after 7 days of osteogenic differentiation of human BMSCs (Zhang et al., 2017). Their results showed that DEGs were enriched in GO terms including cell adhesion and skeletal system development and pathways including cytokine-cytokine receptor interaction, ECM-receptor interaction and TGF-beta signaling pathway, which is consistent with the results of our study. There are few studies systematically analyzed RNA expression changes during different stages of osteogenic differentiation. This study analyzed the transcriptome changes of chicken BMSCs differentiation into osteoblasts at different time point in detail, providing research basis and experimental data for the future study on the osteogenic differentiation process.

The surface markers of BMSCs from different sources differ. CD29 is a member of the integrin gene superfamily that participates in cell-matrix and cell-cell adhesion as an integrin receptor (Jiménez-Marín et al., 2000). CD44 is a multi-structural and multifunctional cell surface glycoprotein that is involved in cell proliferation and differentiation, the docking of proteases on cell membranes, and signal transmission for cell survival (Naor et al., 2002). CD44 provides growth anchor sites for MSCs owing to the binding sites for type I collagen and fibronectin (Bai et al., 2013). CD71 is also known as a marker of BMSC cytoplasts and mediates cellular iron uptake. CD45 is a hematopoietic cell-specific tyrosine phosphatase that is expressed in all leukocytes (Tchilian & Beverley, 2006). RT-PCR assays showed that chicken BMSCs in this experiment were CD29^−^, CD44^−^, and CD71-positive, but CD45-negative, which is consistent with the findings of previous studies (Bai et al., 2013; Bhuvanalakshmi et al., 2014). BMSCs have a vast differentiation potential and are easily accessible in chicken. With the continuous in-depth study of isolation methods and differentiation potential, BMSCs can provide prospects for future research on avian developmental biology and molecular breeding.

Bone characteristics have become an important index for measuring poultry quality during breeding at home and abroad. Osteogenesis is a complex physiological process that has not yet been fully elucidated, especially in poultry. Understanding the process of BMSC differentiation into osteoblasts might help reveal the mechanisms, key genes, and pathways involved in osteoblast differentiation. In this study, BMSCs from White Leghorns were isolated and cultured to differentiate into osteoblasts. Many DEGs associated with osteogenic differentiation were analyzed using transcriptome sequencing of cells collected at different osteogenic differentiation time points. The key gene sets of the different differentiation times were further explored using WGCNA analysis and found to be enriched in several pathways, including DNA replication, cell cycle, Wnt signaling pathway, TGF-β signaling pathway and ECM-receptor interaction. Bones are dynamic organs and are replaced with newly deposited bone in humans and other vertebrates as they age or get damaged (Boskey, 2006). In this study, the DEGs of BMSCs during osteogenic differentiation were partially enriched in GO terms involving the cell surface, such as extracellular matrix, cell surface and cell adhesion, and vascular-related GO terms, including angiogenesis and blood vessel development. Bone marrow contains complex vascular networks and a variety of cell types including hematopoietic stem cells and MSCs. Stromal cells regulate hematopoietic cells by establishing highly specific interactions through niches (Gomariz & Helbling, 2018). BMSCs are found in perivascular locations, and blood vessels play an essential role in maintaining tissue functions, especially for the survival of stem cells within tissues (Sivan et al., 2019).

Genes enriched in DNA replication and the cell cycle may also influence the process of osteoblastic differentiation. Minichromosome maintenance proteins (MCMs) are DNA-dependent ATPases that are loaded onto replication regions and allow them to support the initiation of DNA replication (Sedlackova et al., 2020). The MCM complex consists of six subunits, MCM2–7. MCM3, which serves as a licensing factor, is essential for the initiation of DNA replication in eukaryotic cells. Yamamoto et al. reported that low-level laser irradiation may influence cell proliferation of osteoblasts through the enhancement of MCM family gene expression, such as *MCM3* gene expression (Yamamoto et al., 2001). CDK2 is a catalytic subunit of the cyclin-dependent protein kinase complex that is essential for the G1/S phase of the cell cycle (Fei et al., 2010). Osteogenic growth peptide, one of the stimulators of bone formation, stimulates MSC proliferation and increases the expression of cyclin A in MSCs at both the mRNA and protein levels (Fei et al., 2010).

Energy metabolism plays important role in BMSC survival and differentiation. The intrinsic mechanism of BMSC differentiation remains incompletely understood. Glucose and fatty acid metabolism are considerable regulators in BMSC differentiation (Gastel & Carmeliet, 2021). DEGs during BMSC osteogenic differentiation are enriched in GO terms and KEGG pathways including ATP binding, metabolic pathways, and fatty acid metabolism. Huang et al. identified that the inhibition of *HMGCR* increased glycolysis, and observed the alteration in *HMGCR* gene expression during osteoclast differentiation (Huang et al., 2021). HMGCR activity is a key factor in cholesterol biosynthesis and bone formation in rodents (Mundy et al., 1999). Fatty acid levels in the bone microenvironment are correlated with diseases such as periodontitis, osteoporosis, bone fracture, and tumor-associated bone destruction (Bao et al., 2020). Short-chain fatty acids have been proved to play a crucial role in bone metabolism and immune responses in reducing the severity of arthritis in mice (Lucas et al., 2018). Palmitate, a fatty acid, promotes energy production during osteoblast differentiation and accelerates bone formation (Lee et al., 2017). The generation of ATP is mainly completed by β-oxidation of fatty acids in the mitochondria. Therefore, fatty acid synthesis is an effective way to store carbohydrates. Fatty acids are shuttled and stored in lipid droplets in the form of triglycerides or cholesteryl esters in almost all types of cells for later use (Kushwaha et al., 2018). Fatty acids and lipids can be available for producing ATP for osteoblastic requirements through the citric acid cycle (Lee et al., 2017). Studies have shown that osteoblasts absorb fatty acids; however, their mechanism of action has not been clearly described. The expression of *FADS2*, *ACAA2*, *ACSL5*, and *ACAT2*, which are enriched in fatty acid metabolism, was significantly increased at the early stage of osteogenic differentiation. Genetic variation of a few fatty acids at the plasma level and variants in the *FADS1*-*FADS2* gene regions were correlated with the estimated bone mineral density and fracture risk in summary-level data from up to 426,824 individuals in the UK Biobank (Yuan et al., 2020). Evidence on the association of individual fatty acids with bone mineral density and fracture risk in chickens is scarce and is a prospective research direction. Energy metabolism enables BMSCs to match the different demands of osteo-adipogenic differentiation. Additional studies are needed to fully understand the metabolism in osteogenesis.

Wnt proteins are secreted morphogens that are indispensable for basic developmental processes and are conserved in species from nematodes to mammals (Maeda & Kobayashi, 2019). A total of 19 types of Wnt proteins have been identified in humans. Secreted Wnt proteins activate the Wnt signaling pathway in target cells. The β-catenin-mediated canonical Wnt signaling pathway and β-catenin-independent non-canonical Wnt signaling pathways are two types of Wnt signaling pathways. The β-catenin protein is involved in various physiological processes, such as cell adhesion and transcriptional regulation, and interacts with Wnt ligands. Wnt/β-catenin signaling is an important regulator of MSC fate, and Wnt family members Wnt6 and Wnt10a are the most promising candidates for bone formation (Cawthorn et al., 2012; Feng et al., 2020). In our study, *Wnt6* was also one of the DEGs in the late stage of osteogenic differentiation, which is consistent with the results of Cawthorn. et al. The bone mass of transgenic mice overexpressing *Wnt10b* in adipose tissue was increased (Bennett et al., 2005). The role of Wnt signaling in osteogenesis has been extensively studied in mammals and humans.

The TGF-β signaling pathway and the bone morphogenetic protein (BMP) signaling pathway, play multiple roles in cell proliferation, differentiation, adhesion, and senescence (Zhao et al., 2020). To date, more than 20 BMPs have been documented, which are members of the TGF-β superfamily, except for BMP1 (Wozney et al., 1989). The growth factor BMPs mediate a variety of biological processes, such as embryo development and skeletal morphogenesis (Wan & Cao, 2005). *BMP2*, one of the DEGs involved in late osteogenic differentiation in our study, is related to skeletal development, postnatal bone growth, and fracture repair. TGF-β receptor II (*TGFBR2*) is also necessary for postnatal bone development in osterix-expressing cells (Peters et al., 2017). *BMP3* can act as an endogenous antagonist of the BMP signaling pathway in bone formation owing to its similarity and abundance of amino acids in the bone extracellular matrix (ECM) (Lowery & Rosen, 2018). Collagen type IV alpha 2 (*COL4A2*), a member of the ECM-receptor interaction that is enriched in the late stage of osteogenic differentiation, is the main structural component of the basement membrane. *COL4A2* is likely a potential biomarker in esophageal cancer (Warnecke-Eberz et al., 2016), lung cancer (Ilhan-Mutlu et al., 2016), and breast cancer (Huan et al., 2014). Few studies have clearly described the direct relationship between *COL4A2* and bone development. A previous study showed that an atherogenic diet decreases bone density and increases serum cholesterol levels in male mice. Oxidized low-density lipoprotein increased the size and number of osteoclasts, the expression of *CD36* and liver X receptors (*LXRα*) in osteoclasts, and low-density lipoprotein receptor and *LXRα* in osteoblasts, demonstrating that cholesterol could induce bone loss by increasing the activity of osteoclasts in mice (Sul et al., 2020). Interestingly, we also found that *UCHL1*, *ATP8A2*, *CCK*, and *TRH* were enriched in eating behavior (GO:0042755), a biological process, in the GO term. Dietary habits may cause changes in cholesterol and other substances *in vivo*, thereby affecting bone properties, such as bone mass. In addition, the expansion and survival of BMSCs and osteoblasts isolated from CD36-knockout mice decreased *in vitro*, as did the expression of *RUNX2*, osteogenic gene (*OSX*), bone sialoprotein (*BSP*), and osteocalcin (*OCN*), indicating that *CD36* is essential for sufficient bone metabolism and that it mediates bone formation through osteoblasts (Kevorkova et al., 2013).

The coordinated and precise expression of the genome maintains the normal development of multicellular organisms (Foletta, 1996). Transcription factors regulate the expression of target genes involved in proliferation and differentiation (Sevilla et al., 2001). RUNX2 is the first essential transcription factor guiding MSCs to an osteoblastic lineage and inhibiting their differentiation into adipocytes and chondrocytes (Komori, 2010). The gene expression of *RUNX2* increased gradually in our study, similar to the results of Komori (2008), verifying that RUNX2 keeps osteoblasts in an immature stage with low mineralization. However, further research with the extension of osteogenic induction time of chicken BMSCs is needed to verify whether the expression of RUNX2 is inhibited. The activator protein-1 (AP-1) is a dimeric transcription factor composed of JUN, FOS, and activating transcription factor subunits (Karin et al., 1997). *JUN* and *FOS* were DEGs within the MEturquoise module, and JUN protein exhibited the highest degree value in the PPI network. Prolonged expression of c-FOS results in potentiation of osteoclast differentiation (Miyauchi et al., 1994). FOS and JUN proteins are initially highly expressed and then inhibited during mineralization, which is essential to osteoblast differentiation in mice (Sandberg et al., 1988). Adequate dietary supplementation of calcium, vitamin D, and phosphorus affects bone strength in poultry. The gastrointestinal tract is essential for the absorption of nutrients and health in humans and animals. Distinct FOS/JUN members of gastrointestinal epithelium in the chicken embryonic digestive tract were analyzed by *in situ* hybridization and were important in preserving epithelial–mesenchymal interactions (Matsumoto et al., 1998). Therefore, FOS/JUN proteins, which are well-studied in osteogenic differentiation, can serve as candidate transcription factors affecting bone development in chickens by influencing the absorption of nutrients in intestinal epithelial cells.

In this study, dynamic changes in the transcriptome during osteogenic differentiation of BMSCs in White Leghorns were outlined. The DEGs from the gene set related to cell proliferation, such as cell cycle and DNA replication in the early stage of osteogenic differentiation, to the gene set enriched in the Wnt and TGF-β signaling pathways in the late stage of osteogenic differentiation, were analyzed and discussed. A DEG-related PPI network was constructed using the STRING database. The results of this study detail the transcriptome changes during the differentiation of BMSCs into osteoblasts, as well as the genes and pathways associated with bone development processes, such as osteoblast differentiation and mineralization.

## Data Availability

The datasets presented in this study can be found in online repositories. The names of the repository/repositories and accession number(s) can be found below: NCBI BioProject accession number: PRJNA824833.
